# Fragmentation of the mitochondrial network in skin *in vivo*

**DOI:** 10.1371/journal.pone.0174469

**Published:** 2017-06-23

**Authors:** Daniel Mellem, Martin Sattler, Sonja Pagel-Wolff, Sören Jaspers, Horst Wenck, Michael Alexander Rübhausen, Frank Fischer

**Affiliations:** 1 Center for Free-Electron Laser Science (CFEL), University of Hamburg, Hamburg, Germany; 2 Beiersdorf AG, Applied Biophysics, Hamburg, Germany; Tufts University, UNITED STATES

## Abstract

Mitochondria form dynamic networks which adapt to the environmental requirements of the cell. We investigated the aging process of these networks in human skin cells *in vivo* by multiphoton microscopy. A study on the age-dependency of the mitochondrial network in young and old volunteers revealed that keratinocytes in old skin establish a significantly more fragmented network with smaller and more compact mitochondrial clusters than keratinocytes in young skin. Furthermore, we investigated the mitochondrial network during differentiation processes of keratinocytes within the epidermis of volunteers. We observe a fragmentation similar to the age-dependent study in almost all parameters. These parallels raise questions about the dynamics of biophysical network structures during aging processes.

## Introduction

Cells in human tissue produce their energy in form of adenosine triphosphate (ATP) basically by oxidative phosphorylation. This process takes place in mitochondria, that form highly dynamic networks adapting fast to the cells’ environment and their metabolic requirements. [1] In recent years, numerous experiments have been performed to investigate correlations between mitochondrial network states and corresponding metabolic processes within cells. Cancer cells lacking of a glycolytic medium establish fusion states of the mitochondrial network correlating with a change in energy production going from glycolysis to oxidative phosphorylation. [2] In contrast, a transfer from respiratory to glycolytic conditions leads to a fragmentation of the mitochondrial network. [3] During moderate stress mitochondrial networks form hyperfusion states which coincide with increased ATP production. [4] On the contrary, high stress levels induce to a fragmentation of networks. [5] Mitochondrial fission and fusion states are considered to be quality saving mechanisms of the cell. Fission states support repelling of heavily damaged mitochondria from the network, e.g. during autophagy and mitophagy. [6, 7] In contrast, fusion states help to compensate for defect protein complexes or rare metabolites among mitochondria. [8, 9] Recently, a fragmentation of the mitochondrial network with age was observed *in vitro*. [10] The interplay of mitochondrial dynamics was simulated biophysically in a probabilistic quality model which also revealed a significant fragmentation of the mitochondrial network during aging. [11]

In this paper, we present investigations of mitochondrial morphologies during aging and differentiation of keratinocytes in human skin *in vivo*. We performed two studies: The first study explored variations of the mitochondrial network in epidermal keratinocytes of young and old volunteers. The second study extended the results by investigating the role of the mitochondrial network during the epidermal turnover.

## Materials and methods

**Fig 1:**
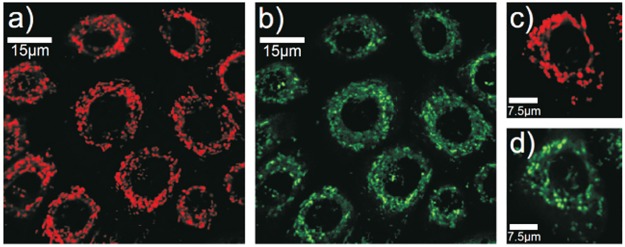
Fluorescence of dyed mitochondria (a) and autofluorescence of NADH (b) in keratinocytes (zoom 3.0). Fluorescence of dyed mitochondria (c) and autofluorescence of NADH (d) in a single keratinocyte (zoom 7.9).

Tissue can be investigated non-invasively *in vivo* by imaging endogenous fluorophores using multiphoton microscopy. [12–14] An important metabolic fluorophore in the epidermis is nicotinamide adenine dinucleotide (NADH) [15, 16] which serves as an electron carrier from the Krebs cycle to respiratory chain. Thus, NADH is almost exclusively located in mitochondria. We tested this premise by *in vitro* investigations using a confocal scanning microscope (Leica TCS SP5, Leica Microsystems, Mannheim, Germany). Neonatal human epidermal keratinocytes were purchased from Lonza Group AG (Basel, Switzerland) and cultured in Dulbecco’s Modified Eagle Medium (DMEM) (Life Technologies, Carlsbad, USA), supplemented with 10% fetal calf serum (PAA Laboratories, Pasching, Austria), L-glutamine and penicillin/streptomycin (both: Life Technologies, Carlsbad, USA). During culturing cells were maintained in a humidifying incubator with a 5% CO_2_ atmosphere at 37°C. Mitochondria in the cells were marked with MitoTracker Red CMXRos (Life Technologies, Carlsbad, USA). We correlated the fluorescence of the dye from 570nm to 650nm to the autofluorescence of keratinocytes in the NADH emission spectrum from 410 nm to 540 nm (Fig 1). The measurements were performed with a 63x objective (HCX PL APO lambda blue 63.0x1.20 WATER UV, Leica). Single images of 2048*2048 pixels were generated with a bidirectional scan either at zoom 3.0 (82*μ*m x 82 *μ*m, step size: 0.04 *μ*m) with scanning rate of 100Hz or at zoom 7.9 (31.2*μ*m x 31.2*μ*m, step size: 0.04*μ*m) with scanning rate of 200Hz.

We found a nearly perfect agreement of morphologies of stained mitochondria with the autofluorescence signal of NADH. Aberrations are possibly caused by an overstaining of the dye, leading to very high intensities and consequently to agglomerations of mitochondrial morphologies in the micrograph. Intensity deviations among autofluorescent mitochondrial structures are assumed to be fluctuations in the metabolic activity. Previous studies indicate that the concentration of NADH is five times higher [17] and the quantum yield is 1.5 to 2.5 greater than of coenzyme NADPH [18] that is fluorescing in the same spectral range. So, potential interference of NADH with the coenzyme NADPH can be neglected.

Imaging mitochondrial network morphologies *in vivo* by the autofluorescence of NADH was performed with a multiphoton microscope (Dermainspect, developed by Jenlab GmbH (Jena, Germany) in collaboration with Beiersdorf AG (Hamburg, Germany) (S1 Fig). The device includes a femtosecond laser with a repetition rate of 80MHz and a pulse width of 150fs. Laser pulses excite the autofluorescence of NADH in the epidermis at a wavelength of 750nm. The autofluorescence is separated from the laser light by a 548nm ± 150nm band-pass filter (HQ 548/305m-2P, Schott AG, Mainz, Germany) and detected by a photomultiplier tube (PMT). In order to image a single cell layer in the epidermis, the focus of the laser through an objective (40x magnification, 1.3 numerical aperture) is scanned parallel to the surface of the skin with an image acquisition time of about 12,5s. During the measurement the z-position is controlled by a piezo element. To prevent disturbance in the images by the movement of the volunteer, the objectives adheres to the skin of the volunteers. Each image has the dimensions of 110*μ*m x 110*μ*m and a resolution of 512pixels x 512pixels. In all measurements the laser power after the objective was set to 20 mW. For the quantitative analyses of mitochondrial morphologies we developed an image binarization algorithm that extracted mitochondrial structures from multiphotonic measurements: Firstly, the cytoplasm of each keratinocyte was taken from the measurements by manually annotating the cell borders and the corresponding nuclei in all images. Then, an Otsu-based [19] image algorithm extracted mitochondrial morphologies using binarization (S1 Fig). A pixel in one annotations had to exceed two thresholds in order to be categorized as NADH-autofluorescence during the binarization process: A global threshold which factors the gray values of all pixels in the annotation and a local threshold which is defined by the gray value of the surrounding pixels. Both of these thresholds were calculated via the Otsu-method. The algorithm calculates gradually all possible binarizations and selects the gray value as the optimal threshold that minimizes the Otsu variance:

$$\begin{array}{c} \sigma_{\omega }\left(T\right)=\omega_{\text{white}}\left(T\right)\sigma_{\text{white}}^{2}\left(T\right)+\omega_{\text{black}}\left(T\right)\sigma_{\text{black}}^{2}\left(T\right) \end{array}$$ (1)

In the Eq (1) *T* represents the threshold of the gray value for the binarization, *σ*_*ω*_(*T*) its Otsu variance, $\sigma_{i}^{2}\left(T\right)$ the variances of the gray values of black and white pixels and *ω*_i_(*T*) their corresponding weights. After binarization procedures, the algorithm assigns all signal related pixels to common signal clusters, so that they fulfill the definition of 4-neighbor-connectivity. The number of pixels per cluster defines its area *A*, the number of edges which border on black pixels (the background of the measurement) defines its circumference *U* and the parameter *C* = *U*^2^/4*πA* defines its circularity. *A* represents the volume of a mitochondrial structure, *C* its circularity. Mann-Whitney-Tests were used for statistical analysis (p≤0.05:*; p≤0.01:**; p≤0.001:***). The analysis was performed with the software “Statistica” by StatSoft (Europe) GmbH (Hamburg, Germany). All corresponding plots were depicted with the software OriginPro 8 (OriginLab Corporation, Northampton, USA).

Two clinical *in vivo* studies were performed. Written informed consent was obtained from each volunteer in both studies. All volunteers provided skin without pathologies and possessed skin types II and III of the Fitzpatrick phototyping scale. [20] Prior and during the studies all volunteers were required to desist from visits of solariums and intensive sun exposure. Treatment of skin in the investigated areas with cosmetic substances and medicals was prohibited during the studies. Measurements in four areas at the inner side of the forearm were performed. In each area multiphotonic images were acquired at three positions. The results of all positions were averaged in each area. The examinations were performed by trained personnel at standard atmospheric conditions (23°C ± 1°C and 43% ± 2% relative humidity). All volunteers were provided with fifteen minutes of acclimatization prior to the measurements. The studies were conducted according to the recommendations of the current version of the Declaration of Helsinki and the Guideline of the International Conference on Harmonization Good Clinical Practice, (ICH GCP). In addition, this study was approved and cleared by the institutional ethics review board (Beiersdorf AG, Hamburg, Germany). Written informed consent was obtained from each volunteer in both studies.

## Results

**Fig 2:**
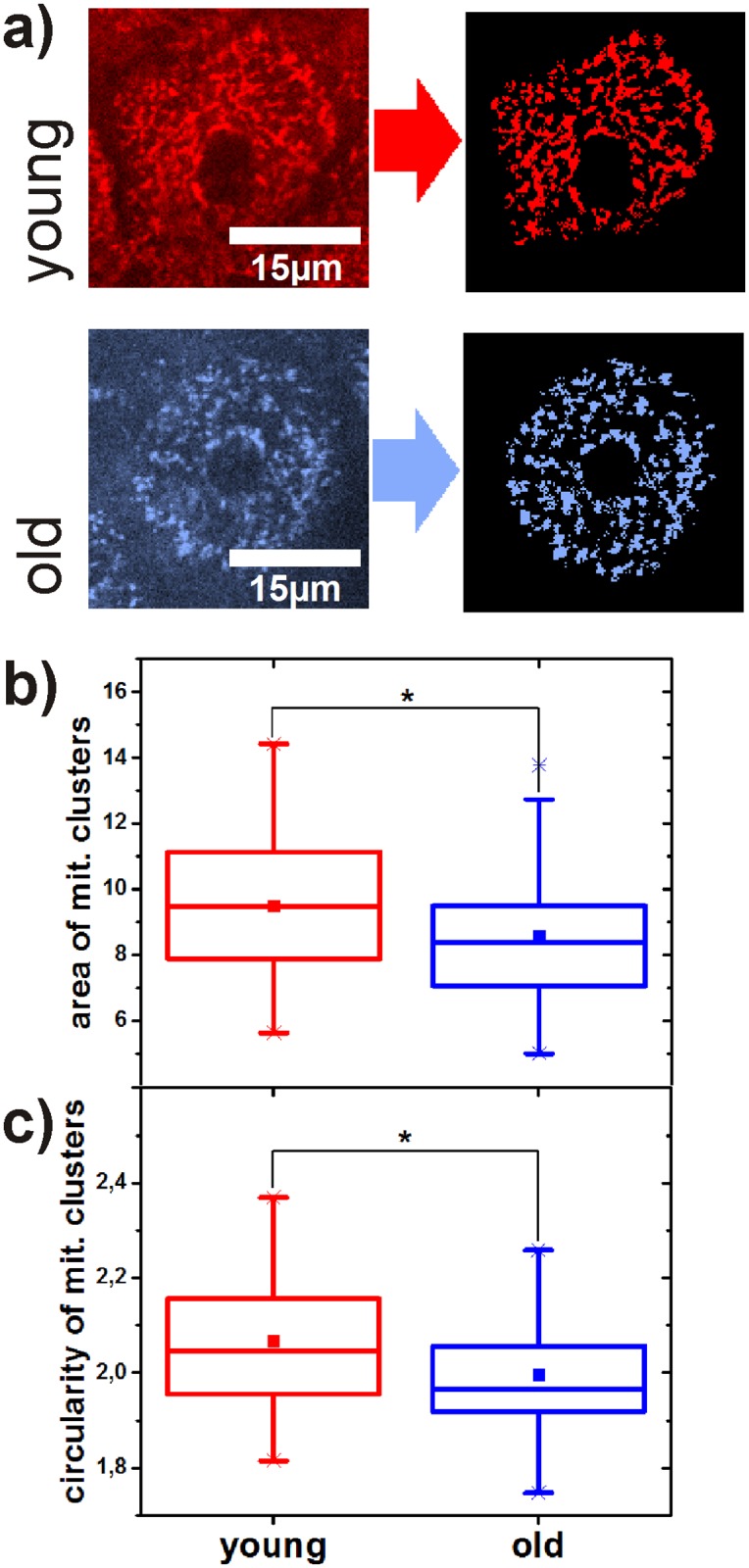
(a) Comparison of mitochondrial networks in young volunteers and old volunteers before and after binarization. Age-related comparison of size (b) and circularity (c) of mitochondrial clusters in keratinocytes. The p-value represents the probability if the two compared probability distributions derive from the same population. The square in the box represents the mean value, the horizontal line in the center the median. 25% percent of the measured values are lower than the bottom of the box, 75% of them are lower than the top of the box. The antennas (whiskers) are the corresponding limits for 2.5% and 97.5%. Crosses represent the highest and the lowest value in the distribution.

In the first study the mitochondrial morphology in epidermal keratinocytes of twelve young (mean ± SD: 23.75 ± 1.67 years) and twelve old (72.17 ± 4.15 years) volunteers was analysed by statistically comparing 48 areas in each age group. We analysed the number, area and circularity of the mitochondrial clusters per keratinocyte in the stratum granulosum in a depth of about 15 *μ*m. The results of all examined parameters were averaged in each area before statistical comparison. Mitochondrial clusters in the stratum granulosum of young volunteers are significantly larger (p = 0.029) and have a significantly higher circularity (p = 0.014) (Fig 2). In contrast, the number of mitochondrial clusters normalized to the size of each keratinocyte is significantly higher (p = 0.006) in old skin (S2 Fig).

**Fig 3:**
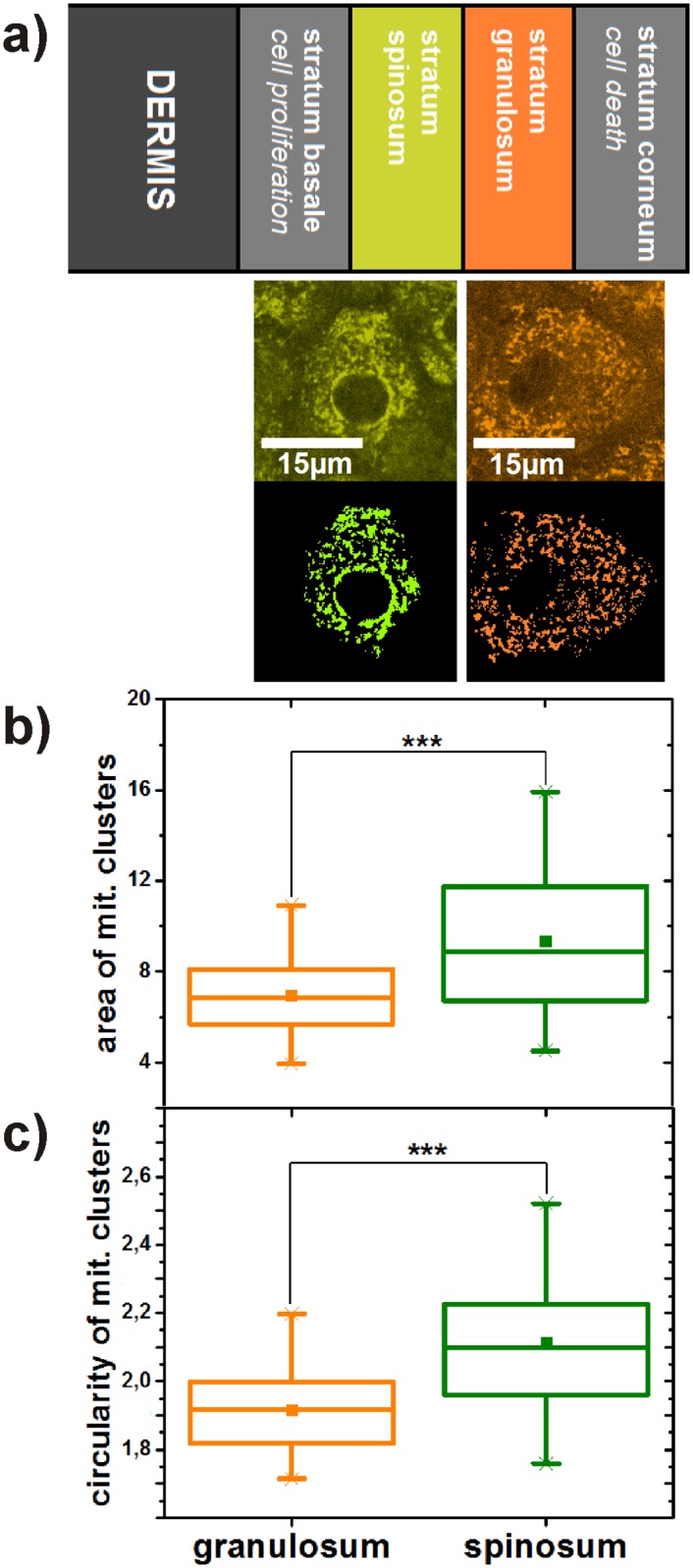
(a) Comparison of mitochondrial networks in stratum spinosum and stratum granulosum representing different states during the differentiation process of keratinocytes. Differentiation-related comparison of size (b) and circularity (c) of mitochondrial clusters in keratinocytes. The p-value represents the probability if the two compared probability distributions derive from the same population. The square in the box represents the mean value, the horizontal line in the center the median. 25% percent of the measured values are lower than the bottom of the box, 75% of them are lower than the top of the box. The antennas (whiskers) are the corresponding limits for 2.5% and 97.5%. Crosses represent the highest and the lowest value in the distribution.

In the second study the morphology of the mitochondrial networks in keratinocytes of the stratum spinosum (depth of about 25 *μ*m) and the stratum granulosum (depth of about 15 *μ*m) were analysed by statistically comparing 32 areas in each layer. For determination of the layers, a prescan through the epidermis was performed during which the mosaic structure of the spinolar cells and the larger and rounder granular cells were identified by trained personnel. All eight volunteers were of the same age group (62 ± 1.31 years). Again, investigated parameters were averaged in each area. The average size per mitochondrial cluster is significantly larger (p ≤ 0,001) and its circularity significantly higher (p ≤ 0,001) in the stratum spinosum (Fig 3). Contrary, the number of mitochondrial clusters normalized to the cell size tends to be higher (p ≤ 0,086) in the stratum granulosum (S3 Fig).

## Discussion

Our study of the mitochondrial network during aging reveals, that granular epidermal keratinocytes in young skin have less clusters in total than keratinocytes in old skin. Additionally, clusters in granular keratinocytes of young volunteers are bigger in size and less circular in shape. These findings reveal a high connectivity among mitochondria in the stratum granulosum in young skin and a fragmented mitochondrial network in the stratum granulosum of old skin. Granular keratinocytes of young volunteers seem to prefer a fusion state of the mitochondrial network which is not maintained during aging.

In our second study we investigated the changes of mitochondrial morphologies during epidermal differentiation. At the beginning of the epidermal turnover proliferation of keratinocytes occurs in the deepest layer of the epidermis, the stratum basale. During their differentation process keratinocytes migrate to upper layers, i.e. the stratum spinosum and the stratum granulosum, and finally transform into dead corneocytes in the stratum corneum. [21] For an analysis of mitochondrial network states during differentiation, we performed examinations in two layers above the stratum basale: in the stratum spinosum and the stratum granulosum. The stratum spinosum is the layer next to the stratum basale, so that keratinocytes in the stratum spinosum are less differentiated than in the stratum granulosum which is the last living epidermal layer before cornification occurs. We observed that the number of mitochondrial clusters tends to increase during differentiation. Additionally, clusters significantly shrink to smaller volumes and more compact shapes. These parameters indicate that mitochondrial networks in the stratum spinosum establish fused states which fragment during differentiation processes.

Comparing the results of the two studies the dynamics in mitochondrial network morphologies during differentiation agree well with changes during skin aging in volunteers. In the following we refer to several processes which play a role in the fragmentation of the mitochondrial network.

Reactive oxygen species (ROS) are produced due to long-term solar UV radiation [22] as well as intrinsic generation by mitochondria and other cell organelles. [23] Oxidative stress causes long-term damage to intramitochondrial structures [24] and leads to mitochondrial fission states during aging. [25] Numerous experiments point to the importance of oxidative stress for differentiation processes of keratinocytes. [26] The exclusion of heavily ROS-damaged mitochondria from the mitochondrial network could thereby lead to a fragmentation of the network’s morphology during aging and differentiation.

Mitochondrial aging processes lead to a quality decay of mitochondria [27] which correlates with an age-related decrease of mitochondrial membrane potentials (MMP). [28] The decrease of the MMP also plays a role during the differentiation of keratinocytes. [21] Experiments revealed that mitochondria with low MMPs are excluded from the network by autophagic processes. [29]

Mitophagic and autophagic processes exclude heavily damaged mitochondria from the network. Mitochondrial autophagy increases during the aging of cells [30], thereby fragmenting intermitochondrial connections. Autophagic activity is similarly involved in the differentiation of keratinocytes. [31]

In cultivated keratinocytes an increased glycolytic activity in old volunteers was observed. [32] This shift from oxidative phosphorylation to glycolysis during aging correlates with a fragmentation of the mitochondrial network. Recent publications disclose the change of mitochondrial metabolisms during the differentiation process. [33]

There are several alternatives to explain the similarities of mitochondrial morphologies during aging and differentiation. For instance, keratinocytes could exploit natural aging processes to catalyze differentiation procedures which consequently leads to a fragmentation of the mitochondrial network. Another possible explanation is that mitochondrial morphologies are independent from differentiation and purely influenced by aging processes during the epidermal turnover. Further investigations are necessary to clarify the physiological connections between aging and differentiation regarding mitochondrial morphologies in cells. The influence of aging processes on mitochondrial quality was investigated in computational quality models. [11, 34] A fragmentation of the mitochondrial network during aging of cells and a deceleration of fission and fusion cycles as a quality saving process were observed. The models reveal quality saving mechanisms for mitochondria such as the interplay of mitochondrial networking and recycling and the quality saving benefit of a decreased mitochondrial repair ability in old cells. Hence, our findings are qualitatively in good agreement with the model.

## Conclusion

In conclusion, we have measured and analyzed the morphology of mitochondrial networks in human skin *in vivo* for the first time. We found a significant fragmentation of mitochondrial morphologies in granular keratinocytes during aging. Our results are qualitatively in good agreement with age-dependent investigations *in vitro* and with simulations of biophysical models. Moreover, we observed a fragmentation of the network during the differentiation process of keratinocytes in the epidermis. The parallels of both studies raise questions about the linkage of aging and differentiation concerning mitochondrial morphologies.

## Supporting information

S1 Fig (a) setup of multiphoton microscope (b) measurement of autofluorescence of NADH in stratum granulosum (c) binarization of measured keratinocyte with otsu algorithm. (TIF)

S2 Fig Comparison of number of mitochondrial clusters in young and old volunteers normed to the size of keratinocytes. (TIF)

S3 Fig Comparison of number of mitochondrial clusters in stratum spinosum and stratum granulosum normed to the size of keratinocytes. (TIF)

S1 Dataset The .csv-file provides the data of mitochondrial cluster numbers, cluster size and cluster circularity in all 40 areas that were investigated in the skin of young and old volunteers and in the stratum spinosum and the stratum granulosum. (CSV)
