# Supplementary figures and images for: Fragmentation of the mitochondrial network in skin in vivo
Source: PLoS One. 2017 Jun 23;12(6):e0174469. doi: 10.1371/journal.pone.0174469 (PMC5482427; doi:10.1371/journal.pone.0174469)

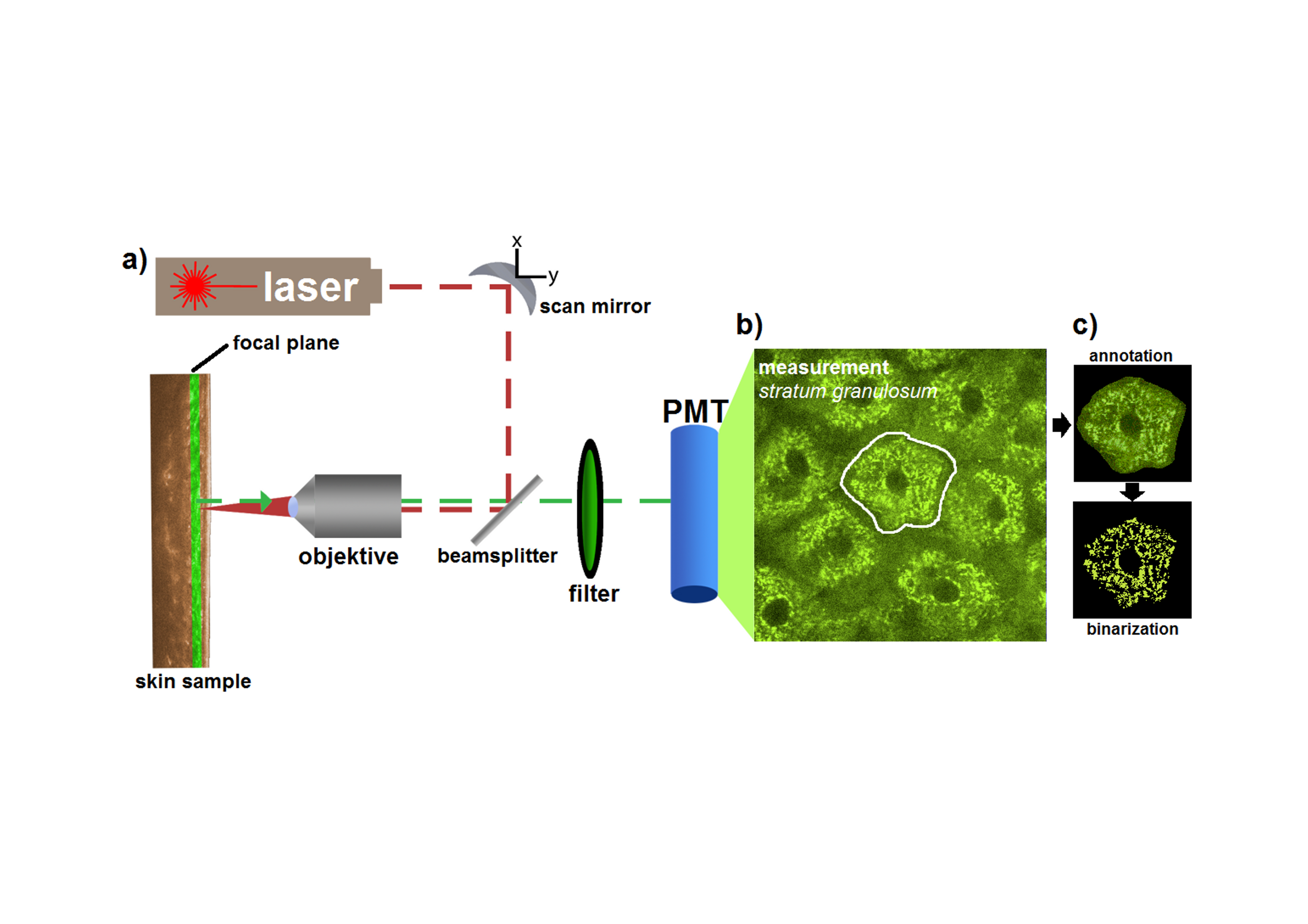

Supplement: S1 Fig — (TIF) [file pone.0174469.s001.tif]

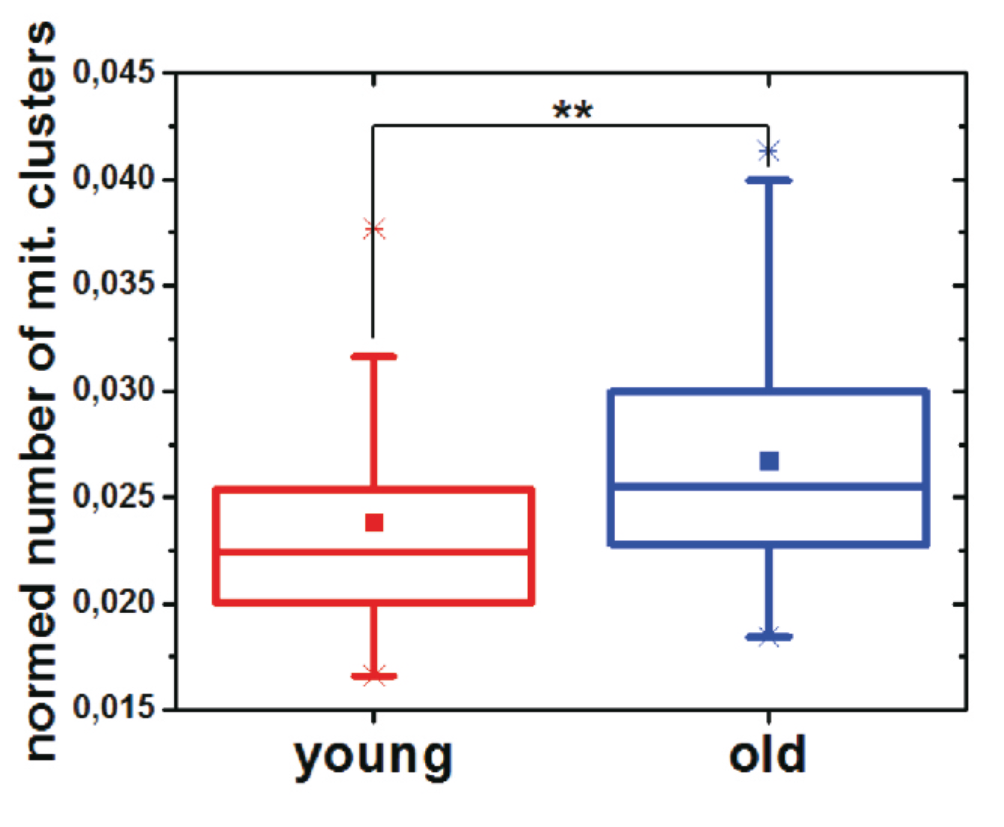

Supplement: S2 Fig — (TIF) [file pone.0174469.s002.tif]

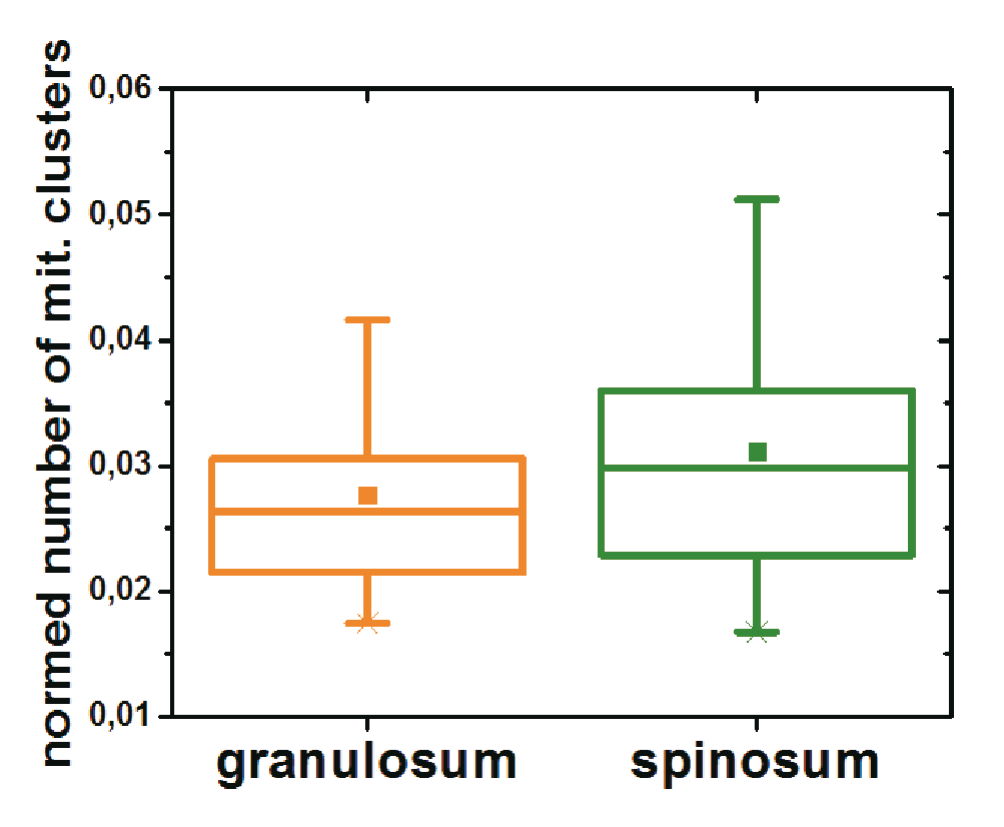

Supplement: S3 Fig — (TIF) [file pone.0174469.s003.tif]
